# Positive Feedback Stimulation of *Ccnb1* and *Mos* mRNA Translation by MAPK Cascade During Mouse Oocyte Maturation

**DOI:** 10.3389/fcell.2020.609430

**Published:** 2020-11-13

**Authors:** Lan-Rui Cao, Jun-Chao Jiang, Heng-Yu Fan

**Affiliations:** Life Sciences Institute, Zhejiang University, Hangzhou, China

**Keywords:** mRNA translation, polyadenylation, kinase, oogenesis, cell cycle, 3′-UTR

## Abstract

In mammalian species, both the maturation promoting factor (MPF) and the mitogen-activated protein kinase (MAPK) cascade play critical roles in modulating oocyte meiotic cell-cycle progression. MPF is a critical heterodimer composed of CDK1 and cyclin B1. Activation of MPF and ERK1/2 requires the activation of maternal *Ccnb1* and *Mos* mRNAs translation, respectively. The phosphorylation and degradation of CPEB1 that triggered by ERK1/2 is a principal mechanism of activating maternal mRNA translation. However, the interplay of these two key kinases in mediating mammalian translational activation of cytoplasmic mRNAs during oocyte maturation is unclear. We prove evidence that the translational activation of *Ccnb1* transcripts containing a long 3′-UTR during meiotic resumption works in an ERK1/2-dependent way. A low level of ERK1/2 activation was detected prior to meiotic resumption. Precocious activation of MAPK signaling in germinal vesicle stage oocytes promotes the translation of *Ccnb1* mRNA and meiotic maturation. Inhibition or precocious activation of CDK1 activity has an appreciable effect on the translation of *Ccnb1* mRNA, suggesting that both kinases are required for *Ccnb1* mRNA translational activation. CDK1 triggers phosphorylation, but not degradation, of CPEB1 in oocytes; the degradation of CPEB1 was only triggered by ERK1/2. Moreover, the translational activation of *Mos* mRNA is regulated by ERK1/2 and cytoplasmic polyadenylation elements too. Taken together, the cooperation and positive feedback activation of ERK1/2 and CDK1 lead to the fine-tuning of mRNA translation and cell-cycle progression during mouse oocyte maturation.

## Introduction

In mammals, resumption of oocyte meiosis occurs after the billow of luteinizing hormone (LH) or upon the mechanistic set free of the oocyte following culture in a proper medium (Edwards and Steptoe, 1975; Komrskova et al., 2014). Germinal vesicle breakdown (GVBD) is the morphological marker of meiotic restoration (Pan and Li, 2019). Following GVBD, meiosis I is accomplished by discharging a polar body-1 (PB1) containing a cluster of chromosomes. The oocytes subsequently get into meiosis II but blocks in metaphase II (MII) until fertilization (Jones, 2011). Full-blown oocytes are transcriptionally silencing and *de novo* protein synthesis of maturing oocytes are depend on post-transcriptional regulation of stored mRNAs, which is mainly mediated by the recruitment of particular transcripts in a patiotemporal manner for translational activation (Chen et al., 2011).

Specifically, meiotic resumption includes the synthesis of necessary key components for meiosis, including cyclin B1 and B2, known as CDK1 kinase regulatory subunit (Han et al., 2017). The metaphase-promoting factor (MPF) are made of CDK1 and cyclin B1, and works as the master regulator of G2-M transition no matter in mitosis or meiosis (Morgan, 1995; Yasuda et al., 1997; Ferrell, 1999). Apart from MPF, the mitogen activated protein kinase (MAPK) cascade also is a critical oocyte meiotic cell-cycle regulator (Fan and Sun, 2004; Liang et al., 2007). Activation of extracellular signal-regulated kinase-1 and -2 (ERK1/2), the most researched family members, is irreplaceable for meiotic spindle assembly and the maintenance of MII arrest (Zhang et al., 2015). The upstream kinases of ERK1/2 are MAPK-ERK kinase 1 and 2 (MEK1/2). In mammalian oocytes, the MAPK cascade is activated by an oocyte-specifically expressed MEK kinase known as the Moloney sarcoma oncogene (MOS) (Verlhac et al., 1996). The translationally dormant mRNAs encoding *Ccnb1* and *Mos* are stored in GV stage-arrested oocytes; their polyadenylation and translation into proteins activate both the MPF and MAPK cascade during meiotic maturation (Komrskova et al., 2014; Kalous et al., 2018).

Translational activation of maternal mRNAs as well as cytoplasmic polyadenylation, require a combinatorial code of *cis*-elements, including cytoplasmic polyadenylation elements (CPEs) and poly(A) signals (PASs) (Reyes and Ross, 2016). The cytoplasmic polyadenylation of CPE-containing mRNAs is regulated by CPE-binding protein-1 (CPEB1) in both *Xenopus* and mouse oocytes (Ivshina et al., 2014). Phosphorylation of CPEB1 on several conserved serine/threonine residues by ERK1/2 triggers its partial degradation, switching CPEB1 from a translational inhibiting factor to an activating factor (Mendez et al., 2002; Setoyama et al., 2007). Meiotic maturation and the translation of a string of maternal mRNAs, including *Tpx2*, *Cnot6l*, *Cnot7*, and *Btg4*, are inextricably linked by activating CPEB1. These maternal translational products are needed for meiotic division and maternal-to-zygotic transition (Sha et al., 2017).

On the contrary, previous studies showed that the activation of ERK1/2 is temporally later than that of MPF and the occurrence of GVBD (Kalous et al., 2018). In addition, accumulation of MOS protein around GVBD is a prerequisite for the activation of the MAPK cascade (Chen et al., 2001). Therefore, the mechanism that triggers the initial activation of *Ccnb1* and *Mos* during the onset of meiotic maturation remains unidentified. It also needs to be investigated whether ERK1/2 regulates the translational activities of maternal *Ccnb1* and *Mos* mRNAs that encode upstream proteins leading to ERK1/2 activation during meiotic progression. These long-standing questions were not yet satisfactorily answered, mainly because MPF and MAPK activities intimately affect each other during oocyte meiosis. Experimental stimulation or inhibition of each kinase inevitably causes an increase or decrease in the activity of other kinases (Zhang et al., 2011). For example, some biochemical studies suggest that CDK1 triggers CPEB1 phosphorylation and degradation as well as translational activation of *Ccnb1* and *Mos* in *Xenopus* oocytes (Kim and Richter, 2007). However, inhibition of CDK1 blocked GVBD and resulted in the activation of many other kinases, including MOS and ERK1/2 (Oqani et al., 2017). For this reason, the distinct roles of the MPF and MAPK cascade in regulating CPEB1 activation and maternal mRNA translation were not concluded.

In this study, we specifically activated the CDK1 or MAPK cascade in GV stage-arrested oocytes in mouse, and investigated their effects on the translational activity of *Ccnb1* and *Mos* transcripts while meiotic progression.

## Results

### ERK1/2 Stimulate Translational Activation of Maternal *Ccnb1* mRNAs Having a Long 3′-UTR

**FIGURE 1:**
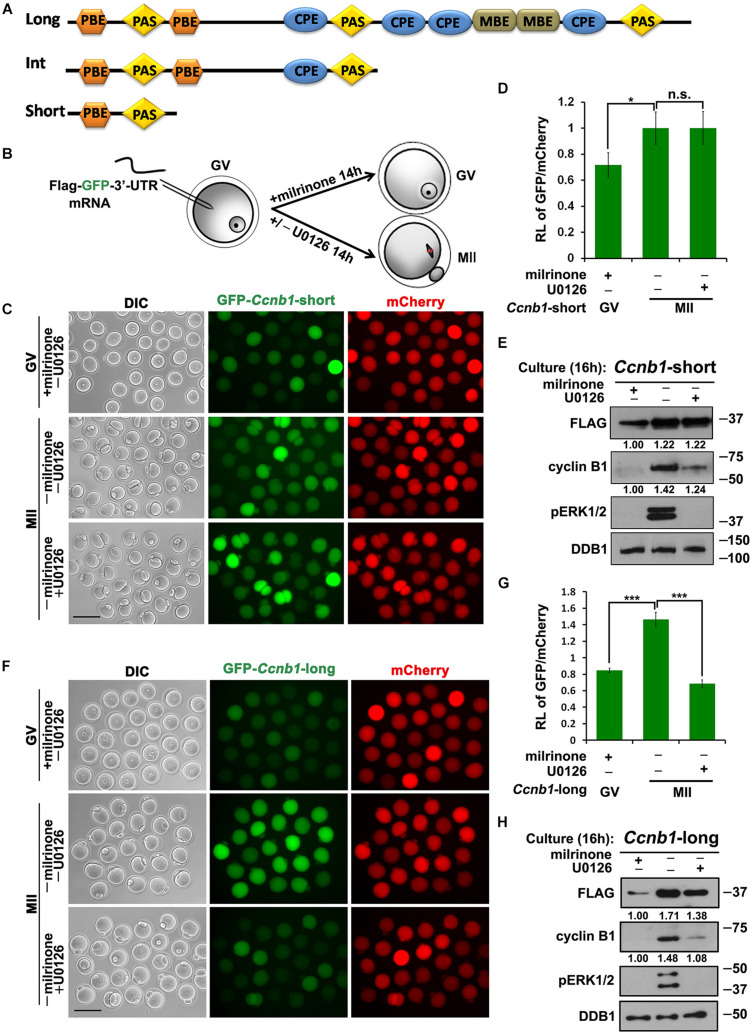
Translational activation of maternal *Ccnb1* mRNAs with short and long 3′-UTRs. **(A)** Schematic representation of three different forms of *Ccnb1* transcripts with distrinct lengths of 3′-UTRs in mouse oocyte. Relative positions of *cis*-elements are indicated. **(B)** An illustration of microinjection and treatments to oocytes in subsequent experiments. **(C,E)** Fluorescence microscopy **(C)** and western blot analysis **(E)** results revealing the expression levels of *Flag*-*Gfp*-*Ccnb1_*short* 3′–UTR_* mRNA in oocytes with different U0126 (20 μM) treatment. DDB1 was used for a control. Numbers under blot bands indicate the intensity of each band. For each set of data, more than 80 oocytes were observed. Scale bar: 100 μm. **(D)** The ratio of the GFP and mCherry fluorescence signals intensity in **(C)**. Data were analyzed by mean ± SEM: *P < 0.05. **(F,H)** Fluorescence microscopy **(F)** and western blot analysis **(H)** data revealing the expression of *Flag*-*Gfp*-*Ccnb1_*long* 3′–UTR_* reporter mRNA in oocytes with or without U0126. For each set of data, more than 80 oocytes were gathered. Scale bar: 100 μm. **(G)** The ratio of the GFP and mCherry fluorescence signals intensity in **(F)**. n.s. indicates non-significant. DDB1 was used for a control. Numbers under blot bands indicate the intensity of each band. Data were analyzed by mean ± SEM: ***P < 0.001.

Three different forms of *Ccnb1* transcripts with different lengths of 3′-UTRs exist in mouse oocytes (Figure 1A; Yang et al., 2017). The accumulation of cyclin B1 proteins during mouse oocyte maturation relies on the translational activation of these transcripts with different 3′-UTRs (Piqué et al., 2008). The *Ccnb1* transcripts with short 3′-UTRs do not contain any CPE and are constantly translated into oocytes during meiotic maturation (Yang et al., 2017). The *Ccnb1* transcripts with intermediate and long 3′-UTRs contain multiple CPEs. Their translation is repressed in oocytes before meiotic resumption, but is activated following GVBD (Tay et al., 2000). In dormant GV oocytes, the resumption of meiosis is possibly driven by the activation of *Ccnb1_*short* 3′–UTR_* mRNA translation. On the contrary, increased synthesis of cyclin B1 by the two other transcripts is important for maintaining active CDK1 during meiotic progression (Kubiak et al., 1993).

In this study, we analyzed whether ERK1/2 activity regulates the activation of *Ccnb1* mRNAs translation during meiotic maturation. We cloned the mouse *Ccnb1* short and long 3′-UTRs and ligated them into the pRK5-*Flag*-*Gfp* vector backbone, which contains an SP6 promoter sequence. Then We *in vitro* transcribed and microinjected *Flag*-*Gfp*-3′-UTR*_*Ccnb*__1_* mRNAs into GV stage oocytes. For this reason, the translational intensity of these transcripts depends upon novelty 3′-UTR*_*Ccnb*__1_*-regulated cytoplasmic polyadenylation (Dai et al., 2019). In this experiment, we *in vitro* transcribed and polyadenylated *mCherry* mRNA, and co-injected reporter mRNA with *mCherry* as a translational standard of reference. Some injected oocytes were released from milrinone-induced GV arrest and cultured in medium with or without the MEK1/2 inhibitor U0126 for 14 h (Figure 1B; Flamigni et al., 1999). After culture, expression of GFP protein was detected in oocytes by epifluorescence and FLAG protein was detected by western blot analyses. The translation activity of the 3′-UTR was quantified by the ratio of the GFP and mCherry fluorescence signals intensity within the same oocyte.

The results implicated that the microinjected *Flag*-*Gfp*-*Ccnb1_*short* 3′–UTR_* mRNA was translated at the GV stage and underwent a modest increase in translation during meiotic maturation after release from milrinone (Figures 1C–E). U0126 blocked the phosphorylation of ERK1/2 and reduced the accumulation of endogenous cyclin B1 during oocyte meiotic maturation (Figure 1E). However, inhibition of ERK1/2 activity in maturing oocytes did not affect the translation of *Flag*-*Gfp*-*Ccnb1_*short* 3′–UTR_* mRNA (Figures 1C–E). These results suggest that the translation of maternal *Ccnb1* mRNAs with the short 3′-UTR is ERK1/2-independent.

In contrast, the translational activity of *Flag*-*Gfp*-*Ccnb1_*long* 3′–UTR_* mRNA was decreased in GV oocytes but increased after meiotic resumption (Figures 1F–H). U0126 treatment reduced the meiotic maturation-coupled translation of this mRNA (Figures 1F–H).

### Activation of ERK1/2 Promotes the Translation of *Ccnb1* mRNA and Induces the Meiotic Maturation

**FIGURE 2:**
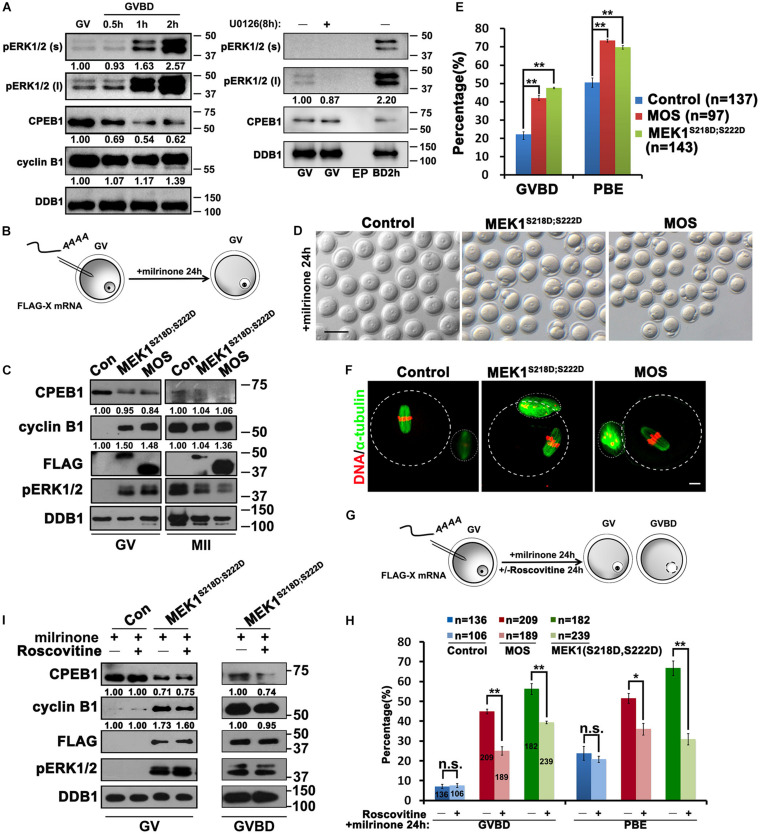
Activation of ERK1/2 promotes the translation of *Ccnb1* mRNA and induces meiotic maturation. **(A)** Western blot analysis results revealing the ERK1/2 phosphorylation and CPEB1 levels at the appointed time points behind meiotic recovery. For each set of data, 100 oocytes were gathered and loaded. DDB1 was used for a control. **(B)** An illustration of microinjection and treatments to oocytes in **(C–F)**. **(C)** Western blot analysis results revealing contents of indicated proteins in oocytes microinjected with mRNAs encoding MOS or constitutively active MEK (MEK1^S218D;S222D^). One hundred oocytes were gathered and loaded in each lane. Numbers under blot bands indicate the intensity of each band. **(D–F)** Representative images **(D)**, GVBD and PB1 emission (PBE) rates **(E)**, and **(F)** immunofluorescence staining results of α-tubulin of oocytes in **(C)**. DDB1 was used for a control. Numbers under blot bands indicate the intensity of each band. The accurate number of oocytes analyzed is labeled (*n*). Data were analyzed by mean ± SEM: **P < 0.05. Dashed lines indicate the oocyte outline. Scale bar: 100 μm. **(G)** An illustration of microinjection and treatments to oocytes in **(H,I)**. **(H)** GVBD and PBE rates in oocytes that overexpressed MOS or MEK1^S218D;S222D^ by microinjection and further cultured in medium containing roscovitine (100 μM) for 24 h. The number of oocytes analyzed is labeled (*n*). Data were analyzed by mean ± SEM: *P < 0.05, **P < 0.01. n.s. indicates non-significant. **(I)** Western blot analysis results showing levels of indicated proteins in **(G)**. For each set of data, 70 oocytes were gathered and loaded. DDB1 was used for a control. Numbers under blot bands indicate the intensity of each band.

Although the activation of ERK1/2 was previously reported to be temporally later than meiotic resumption, western blot results showed that low levels of phosphorylated ERK1/2 (pERK1/2) were detected in fully grown GV oocytes, and the levels of pERK1/2 rapidly increased as soon as GVBD occurred (Figure 2A). This signal of pERK1/2 is specific because it was eliminated in GV oocytes incubated with U0126 (Figure 2A). To further investigate the functional role of ERK1/2 in regulating the translation of *Ccnb1* mRNAs, we specifically activated the MAPK cascade by ectopically expressing a constitutively active MEK1 (MEK1^S218D/S222D^) or the upstream kinase MOS in GV-stage arrested oocytes by mRNA microinjection (Figure 2B; Kalous et al., 2018). In GV oocytes, successful expression of these proteins and precocious activation of ERK1/2 were confirmed by western blot analysis (Figure 2C). Phosphorylation and partial degradation of CPEB1 as well as the translational activation of endogenous *Ccnb1* were also observed in these GV oocytes (Figure 2C). Even in the presence of milrinone in the culture medium, approximately half of the MEK1^S218D;S222D^- or MOS-overexpressing oocytes underwent GVBD (Figures 2D,E). The majority of these oocytes extruded PB1 and reached the MII stage with normal-looking spindles (Figures 2D–F). This result indicated that activation of the MAPK cascade in fully grown oocytes is sufficient to drive meiotic maturation. Meanwhile, activation of ERK1/2 is sufficient for the translational activation of *Ccnb1*, even in oocytes that have intact GVs.

Next, we investigated whether CDK1 activity is required for ERK1/2-triggered translational activation of mRNA and meiosis progression. When the CDK1 inhibitor roscovitine was added to the MEK1^S218D;S222D^ mRNA-injected oocytes (Planchais et al., 1997), PB1 emission was repressed to background levels, but GVBD was only partially inhibited instead of being completely blocked (Figures 2G,H). Furthermore, CPEB1 phosphorylation and partial degradation, as well as the translational activation of *Ccnb1*, was not affected by roscovitine treatment (Figure 2I). These results indicate that the MAPK cascade is sufficient for the translational activation of maternal mRNAs independent of CDK1 activity.

### Activated CDK1 Is Also Required for *Ccnb*1 mRNAs Translational Activation

**FIGURE 3:**
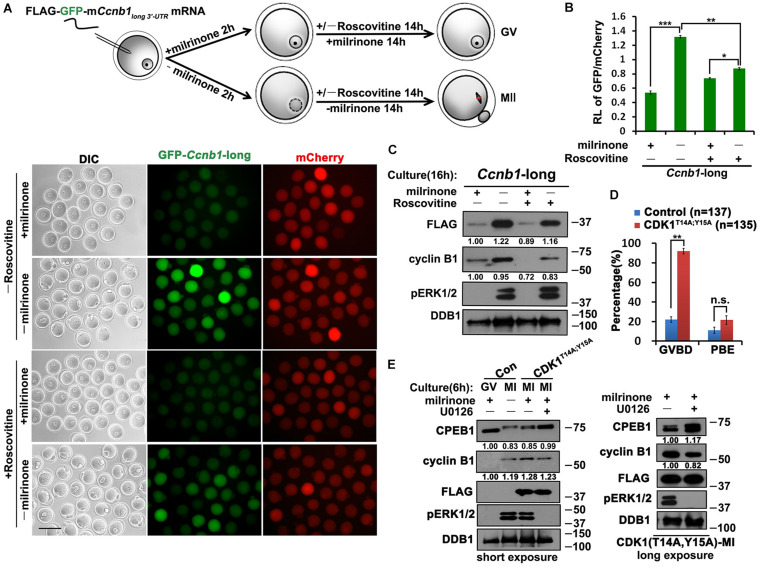
Role of CDK1 in the translational activation of *Ccnb1* mRNAs. **(A)** Fluorescence microscopy results revealing the expression levels of *Flag*-*Gfp*-*Ccnb1_*long* 3′–UTR_* mRNA in oocytes with different roscovitine (100 μM) treatment. For each set of data, 50 oocytes were gathered. Plotting scale: 100 μm. **(B)** Relative fluorescence intensity of GFP relative to mCherry in **(A)**. Data were analyzed by mean ± SEM: *P < 0.05, **P < 0.01, ***P < 0.001. **(C)** Western blot analysis results revealing translational levels of the reporter mRNA as well as endogenous cyclin B1 in **(A)**. For each set of data, 80 oocytes were gathered and loaded. **(D)** GVBD and PBE rates of oocytes microinjected with mRNAs encoding non-inhibitable CDK1 (T14A;Y15A) and cultured for 14 h. The accurate number of oocytes analyzed is labeled (*n*). DDB1 was used for a control. Numbers under blot bands indicate the intensity of each band. Data were analyzed by mean ± SEM: **P < 0.01. n.s. indicates non-significant. **(E)** Western blot analysis results showing levels of indicated proteins in oocytes overexpressed mRNAs encoding CDK1^*T*14*A;Y*15*A*^ and cultured with different milrinone and U0126 (20 μM) treatment. For each set of data, 70 oocytes were gathered and loaded. DDB1 was used for a control. Numbers under blot bands indicate the intensity of each band.

In addition to ERK1/2, we also explored the potential participation of CDK1 in regulating *Ccnb1* mRNA translation during oocyte maturation. We microinjected *Flag*-*Gfp*-*Ccnb1_*long* 3′–UTR_* mRNA into GV oocytes, and further cultured them in a medium with or without the CDK1 inhibitor roscovitine. Roscovitine reduced the translation of reporter mRNA as well as endogenous *Ccnb1* but did not affect ERK1/2 phosphorylation at the MII stage (Figures 3A–C). Consequently, these results suggest that CDK1 activity is also a key link between *Ccnb1* translation and meiotic cell-cycle progression.

Although CDK1 and ERK1/2 kinases are all involved in triggering the translational activation of *Ccnb1*, whether the interplay of these two kinases couples this event to oocyte meiotic maturation remains elusive. Cells can be forced to bypass G2-phase arrest by expressing non-inhibitable CDK1 (CDK1^T14A;Y15A^) mutants (Baldin et al., 1997). Overexpression of CDK1^T14A;Y15A^ in GV oocytes by mRNA microinjection triggered meiotic resumption and promoted the translation of endogenous *Ccnb1* after 4 h (Figures 3D,E). Meanwhile, The lever of CDK1^T14A;Y15A^ induced ERK1/2 phosphorylation is similar to that in control MI oocytes. However, inhibition of ERK1/2 activity by U0126 treatment reduced the levels of cyclin B1 accumulation (Figure 3E). We also observed that CDK1^T14A;Y15A^ triggered the phosphorylation of CPEB1, but CPEB1 could not be degraded after blocking the MAPK cascade (Figure 3E). This result suggests that CDK1 can promote the phosphorylation of CPEB1, but the degradation of CPEB1 depends on ERK1/2 activation.

### Activated ERK1/2 Promotes the Translation of Its Upstream Kinase MOS

**FIGURE 4:**
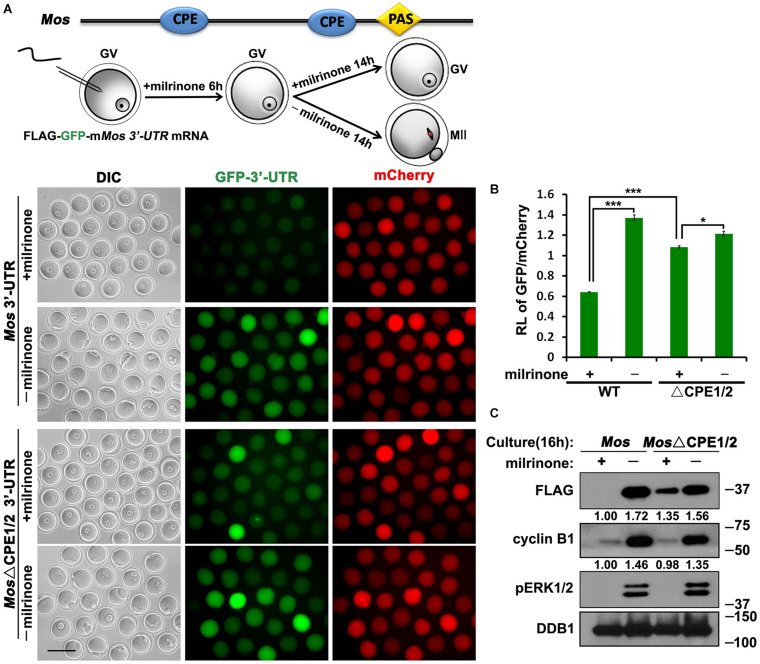
Translational activation of *Mos* mRNA during meiotic maturation in mouse oocytes. **(A,C)** Fluorescence microscopy **(A)** and western blot analysis **(C)** results revealing the expression levels of Flag-GFP fused with *Mos* 3′-UTR or its CPE-mutated (ΔCPE) form. For each set of data, 70 oocytes were observed. Plotting scale: 100 μm. **(B)** The ratio of the GFP and mCherry fluorescence signals intensity in **(A)**. Data were analyzed by mean ± SEM: *P < 0.05, ***P < 0.001. DDB1 was used for a control. Numbers under blot bands indicate the intensity of each band.

Another important cell cycle regulator in mouse oocytes is MOS (Gebauer and Richter, 1997). The corresponding proteins were detected after GV breakdown, and their expression run up to a maximal level in the MII stage oocytes (Gebauer et al., 1994). To study the translation of MOS, we fused WT mouse *Mos* and mutant 3′-UTRs with FLAG-GFP, then these reporter mRNA along with mRNA encoding mCherry expressed in GV oocytes by microinjection. The *Mos* 3′-UTR-driven FLAG-GFP expression was low in GV oocytes but was activated following meiotic maturation (Figures 4A,B). Simultaneous mutations of the two CPEs within the 3′-UTR led to a remarkable increase in the translation of the FLAG-GFP reporter in GV oocytes, but the accumulation of FLAG-GFP did not reach the wild-type levels at the MII stage (Figures 4B,C).

**FIGURE 5:**
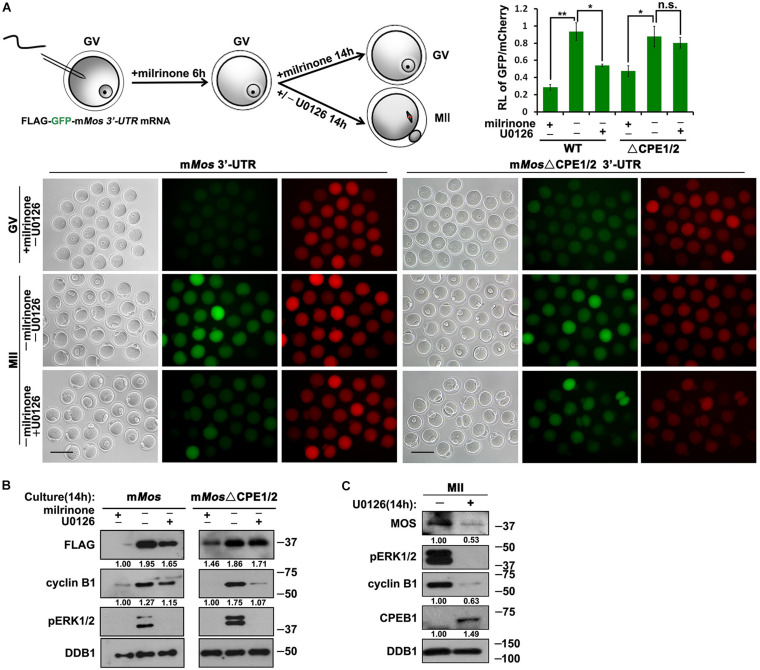
Roles of CDK1 and ERK1/2 in the translational activation of *Mos* mRNA. **(A)** Fluorescence microscopy and the ratio of the GFP and mCherry fluorescence signals intensity results showing Flag-GFP expression driven by WT and CPE-mutated *Mos* 3′-UTR with different U0126 (20 μM) treatment. For each set of data, 50 oocytes were gathered. Plotting scale: 100 μm. **(B)** Western blot analysis results revealing translational levels of the reporter mRNA as well as endogenous cyclin B1 in **(A)**. For each set of data, 80 oocytes were gathered and loaded. DDB1 was used for a control. Numbers under blot bands indicate the intensity of each band. **(C)** Western blot analysis results revealing protein levels of endogenous MOS as well as phosphorylated ERK1/2 expression in mouse MII oocytes with different U0126 (20 μM) treatment. For each set of data, 300 oocytes were gathered and loaded. DDB1 was used for a control. Numbers under blot bands indicate the intensity of each band.

Furthermore, U0126 repressed the meiotic resumption-coupled translational activation of *Flag*-*Gfp*-*Mos_3′–UTR_* (Figures 5A,B). However, the translation of the CPE-mutated reporter mRNA could not be inhibited by U0126 at the MII stage (Figures 5A,B). U0126 also suppressed the accumulation of endogenous MOS during oocyte maturation (Figure 5C). Consequently, these results suggest that activated ERK1/2 promotes the translation of its upstream kinase MOS during meiotic cell-cycle progression by regulating CPE-associated factors.

### ERK1/2 Stimulate the Cytoplasmic Polyadenylation of *Ccnb1_*long* 3′–UTR_* and *Mos* Transcripts

**FIGURE 6:**
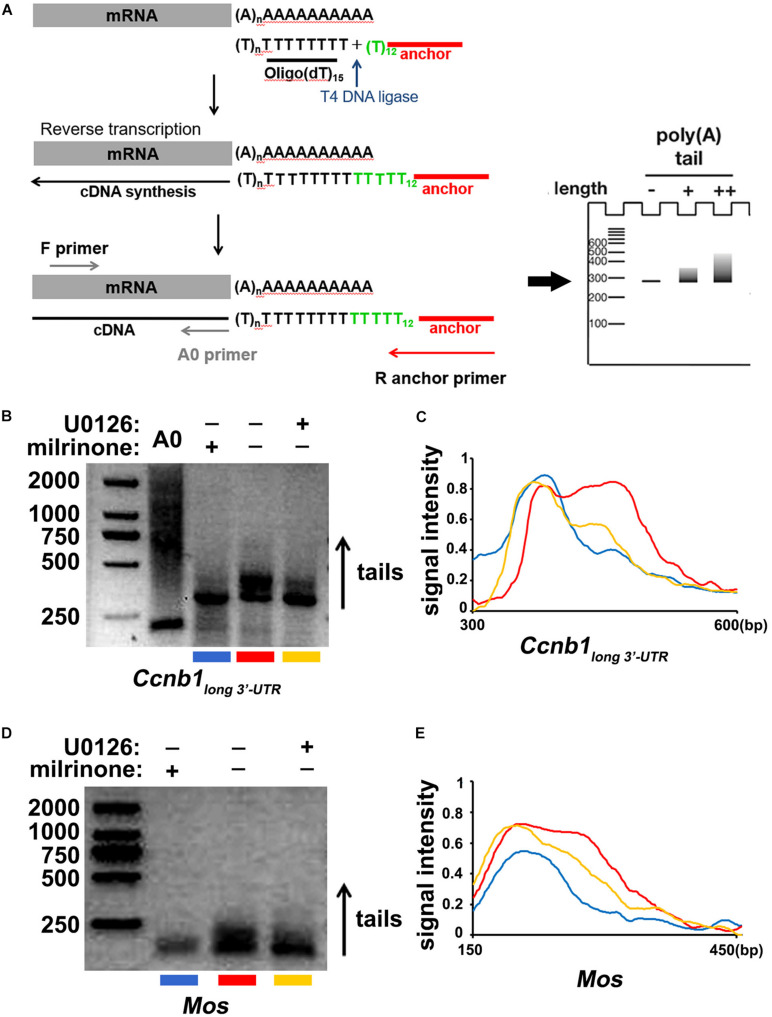
Cytoplasmic polyadenylation of *Ccnb1* and *Mos* transcripts. **(A)** A schematic representation of the poly(A) tail assay (PAT) assay. **(B)** Data of the PAT assay revealing the length of *Ccnb1 _*long* 3′–UTR_* transcripts’ poly(A) tail in oocytes with different U0126 (20 μM) treatment. Conditions of PCR amplification had detailedly described in the section “Materials and Methods.” **(C)** Quantitative statistics of the PAT assay results in **(B)**. The plane coordinate representing the floating length of the PCR products from a appointed point to the *x*-axis, measured relative signal intensity to the *y*-axis. **(D)** Results of the PAT assay revealing the length of *Mos* transcripts’ poly(A) tail in oocytes with different U0126 (20 μM) treatment. **(E)** Quantitative statistics of results in **(D)**.

Finally, we detected the effect of ERK1/2 in regulating the cytoplasmic polyadenylation of *Ccnb1_*long* 3′–UTR_* and *Mos* transcripts. Using a poly(A) tail (PAT) assay (Figure 6A; Sha et al., 2018), we measured the polyadenylation levels of endogenous *Ccnb1_*long* 3′–UTR_* and *Mos* mRNAs after treatment with U0126. The *Ccnb1_*long* 3′–UTR_* and *Mos* transcripts were not significantly polyadenylated at the GV stage (Figures 6B–E). Their polyadenylation levels increased after meiotic maturation in an ERK1/2 activity-dependent manner and were affected by U0126 treatment (Figures 6B–E). These results further indicate that the cytoplasmic polyadenylation of maternal *Ccnb1_*long* 3′–UTR_* and *Mos* mRNA are ERK1/2-induced.

## Discussion

In a previous study, three different *Ccnb1* 3′-UTRs of different lengths were identified in mouse oocytes (Yang et al., 2017). These *Ccnb1* transcripts have different 3′-end polyadenylation and translation dynamics during oocyte maturation. Because the MAPK cascade plays a key role in meiotic resumption-coupled translational activation of maternal mRNAs (Chen et al., 2020), we determined the role of ERK1/2 activity in the translation regulation of *Ccnb1* transcripts with short and long 3′-UTRs. The results indicated that the translation of the *Ccnb1* short isoform is constitutive and ERK1/2-independent. In contrast, the translation of the *Ccnb1* long isoform is GVBD-associated and ERK1/2-dependent. Both the translational repression of the *Ccnb1* long isoform at the GV stage and its translational activation after GVBD relies on the CPEs in its 3′-UTR. Previous studies have shown that ERK1/2 converts the CPE from a translation-repressing element to an activating element by phosphorylating CPEB1 and inducing its degradation. Consistent with these reports, we observed that experimentally induced precocious activation of the MAPK cascade in GV oocytes is sufficient to induce CPEB1 phosphorylation and degradation.

Because previous studies have testified that significant activation of ERK1/2 arises 2 h after GVBD in a rough way in mouse oocytes, CDK1 is considered to be the kinase that phosphorylates CPEB1 and triggers cytoplasmic polyadenylation of mRNAs during meiotic resumption (Kuo et al., 2011). In this study, we detected basal activities of ERK1/2 in the GV stage arrested oocytes, suggesting that the interplays of CDK1 and ERK1/2 are already in action at the beginning of the G2/M transition in mouse oocytes. Furthermore, because CDK1 and ERK1/2 activate each other during meiotic cell cycle progression, it was difficult to distinguish the specific functions of each of these two kinases during oocyte maturation. In this study, we specifically activated CDK1 or ERK1/2 in arrested GV oocytes, then simultaneously repressed the activity of other kinases using specific inhibitors. We observed that the MAPK cascade is sufficient to trigger translational activation of maternal mRNAs, even before meiotic resumption. In addition, although both CDK1 and ERK1/2 can phosphorylate CPEB1, the degradation of CPEB1 is ERK1/2-dependent.

In this study, we only evaluated the role of CPE in the translational regulation of *Ccnb1* and *Mos* 3′-UTR. However, the translational repression and polyadenylation of maternal mRNAs are complicated during mammalian oocyte maturation. In addition to CPEs, Musashi-binding elements (MBEs) and Pumilio-binding elements (PBEs) also exist in the *Ccnb1* long 3′-UTR (Figure 1A), and may determine the transcript-specific spatiotemporal patterns of maternal mRNA translation (Reyes and Ross, 2016). Studies in *Xenopus* have shown that these elements are often regulated by the activities of CDK1 and ERK1/2. For example, Musashi 1 (MSI1) binds its target mRNAs through MBEs and induces the remodling of the RNA structure, therefore revealing neighboring CPEs and stimulating translation during oocyte maturation *in Xenopus* (Weill et al., 2017). In this case, MSI1 can be released from the MBEs in the wake of phosphorylation by RINGO or CDK1. On the contrary, as the downstream of MOS, Nemo-like kinase (NLK) catalyzes the phosphorylation of Pumilio 1 and 2 (PUM1/2) to adjust the translation of cyclin B1 mRNA in *Xenopus* oocytes (Ota et al., 2011).

**FIGURE 7:**
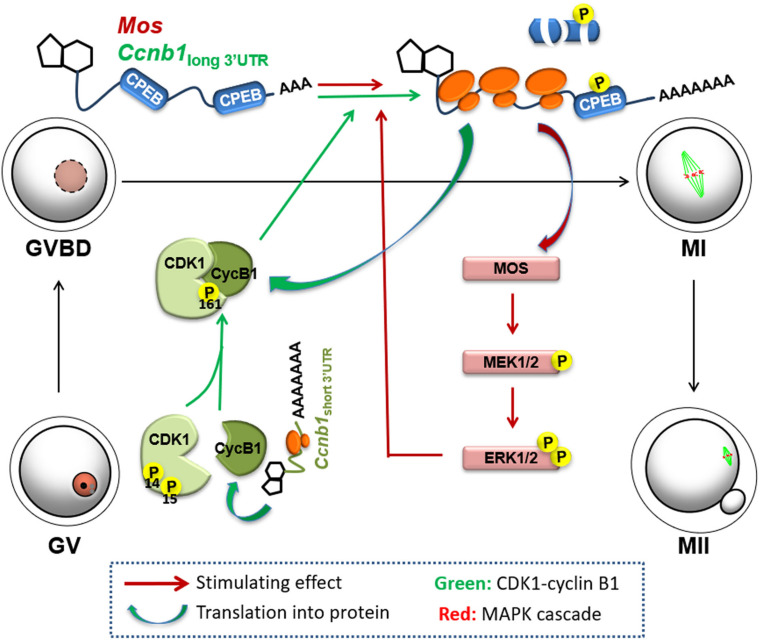
Mechanism of positive feedback stimulation of *Ccnb1* and *Mos* mRNA translation by the MPF and MAPK cascade during mouse oocyte maturation. The short isoform of *Ccnb1* is constitutively translated into cyclin B1 proteins in the GV stage-arrested oocytes, and form a pre-MPF with CDK1. At a threshold timepoint, the basal MPF and ERK1/2 activities trigger translational activation of the *Ccnb1* long isoform and *Mos* mRNAs. Storage of MOS and cyclin B1 brings on a significant increase in CDK1 and ERK1/2 activity, thereby further boosting maternal mRNA translation, forming two entangled positive feedback loops (labeled red and green, respectively), eventually causing meiotic resumption and oocyte maturation.

Based on the results of the current study, we proposed a mechanism of positive feedback stimulation of *Mos* and *Ccnb1* mRNA translation by the MPF and MAPK cascade during mouse oocyte maturation. The short isoform of *Ccnb1* is constitutively translated into cyclin B1 proteins in the GV-stage-arrested oocytes, and form a pre-MPF with CDK1. At a threshold time point, the basal MPF and ERK1/2 activities trigger translational activation of the *Ccnb1* long isoform and *Mos* mRNAs. The accumulation of MOS and cyclin B1 leads to a significant increase in CDK1 and ERK1/2 activity, further boosting maternal mRNA translation and forming two entangled positive feedback loops (labeled red and green in Figure 7), eventually causing meiotic resumption and oocyte maturation.

## Materials and Methods

### Mice

Wild type mice of institute of Cancer Research (ICR) strain were bought from the Zhejiang Academy of Medical Science, China. All mice were kept under SPF conditions in a controllable environment between 20°C and 22°C, with 12 h of light a day, 50–70% humidity in the air, an ample supply of food and water. Animal tendance and experimental methods were performed according to the Animal Research Committee guidelines of Zhejiang University.

### Plasmids Construction and *in vitro* Transcription

Mouse *Ccnb1_*short* 3′–UTR_* and *Ccnb1_*long* 3′–UTR_* cDNAs were obtained by PCR amplification with the following primers: forward primer in common (5′- CTCCAATAGACTGCTACATCTGCAGATG-3′), *Ccnb1_*short* 3′–UTR_* reverse primer (5′-TGAGAAGTCACAACCTTTATTGA AGAGC-3′) and *Ccnb1_*long*__3_*_^´_*_–UTR_* reverse primer (5′-AAAGCT TTCCACCAATAAATTTTATTCAACT-3′). Mouse *Mos_3′–UTR_* was PCR-amplified with primers: FP (5′- CTCCATCGAGC CGATGTAGAGATAAGC-3′) and RP (5′- GAAGTTCGTG GTAACTTTATTTCCTAAAAATATGC-3′). All 3′-UTRs were cloned into pRK5-*Flag*-*Gfp* eukaryote expression vectors and *in vitro* transcribed using the SP6 mMessage mMACHINE Kit (Invitrogen, AM1450) following the user manual. mRNAs settled to the bottom by adding lithium chloride and resuspended with RNase free water. Mouse MEK1 and human MOS coding sequences were cloned into N-terminal FLAG-tagged expression vectors; linearizing vectors were suffered phenol/chloroform extraction and ethanol precipitation for microinjection. The SP6 mMessage mMACHINE Kit and a Poly (A) Tailing Kit (Ambion) were used for *in vitro* transcribing. mRNAs were obtained by adding lithium chloride and dissolving in nuclease-free water.

### Oocyte Collection and Culture

Pregnant mare serum gonadotropin (PMSG, Ningbo Sansheng Pharmaceutical, China) can be used for ovulation. Female mice at 21 days of age were injected 5 IU of PMSG for 44 h, then the mice were humanely euthanized and disposed. Oocytes were collected using M2 medium (M7167; Sigma-Aldrich) and put into M16 medium (M7292; Sigma-Aldrich) for culture, all drops were covered with mineral oil (M5310; Sigma-Aldrich) in a 5% CO_2_ atmosphere at 37°C. In this study, U0126 (20 μM) was added to inhibit ERK1/2 activation of oocytes, and roscovitine (100 μM) was added into the media to inhibit CDK1 activation.

### Microinjection

Full-brown GV oocytes were collected with M2 medium cotaining 2 μM milrinone, which can inhibite spontaneous GVBD. All operation was performed using an inverted microscope (Eclipse TE200; Nikon). Synthetic mRNA diluted in RNase free water was injected into the cytoplasm of GV stage oocytes. Bare oocytes were each injected with 5–10 pl sample per oocyte.

### Immunofluorescence and Confocal Imaging

Oocytes were fixed in 4% paraformaldehyde (PFA), this process lasted 30 min at room temperature. Then oocytes were cultured in 0.1% Triton X-100 that act as dialytic buffer at room temperature for 20 min. After blocking in 1% bovine serum albumin (BSA) in PBST, oocytes were labeled with a FITC-conjugated anti-α-tubulin antibody and 5 μg/ml of DAPI for 30 min. The mounting microscopic glass were fixed on oocytes using SlowFade Gold Antifade Reagent (Life Technologies), and signal acquisition was performed on a Zeiss LSM710 confocal microscope.

### Western Blot Analysis

Protein samples were lysed in β-mercaptoethanol containing cracking buffer and heated at 95°C for 10 min. Then SDS-PAGE was used to separate total protein and transferred via electrophoresis onto PVDF membranes (Millipore, Bedford, MA, United States). Membranes were blocked in 5% non-fat milk (BD, Franklin Lakes, NJ, United States) for 30 min, followed by probing with primary antibodies at 4°C overnight. After washing in TBST three times, membranes were incubated with a secondary antibody (Jackson ImmunoResearch Laboratories) for 1 h. Membranes were washed in TBST three times again. Signals were detected using the Super Signal West Femto maximum sensitivity substrate (Thermo Fisher Scientific, Waltham, MA, United States). The primary antibodies and diluted concentration used are listed in Supplementary Table 1.

### Poly(A) Tail (PAT) Assay

RNeasy Mini kit (Qiagen, 74106) was used to extract total RNA from 150 oocytes at the indicated stages. R (5′-GCGAGCTCCGCGGCCGCGT_12_–3′) was attached to Oligo(dT) by T4 DNA ligase. SuperScript II (Invitrogen) catalyzed the reverse transcription with Oligo(dT)-anchored R. To amplify the endogenous transcripts’ poly(A) tails, a PCR reaction with gene-specific primers (Supplementary Table 2) and the dT anchor primer R were performed. The PCR cycling system was set as follows: 30 s at 94°C, 20 s at 58°C, and 40 s at 72°C. PCR products were loaded on a 2% agarose gel to represent the polyadenylation states, and images were gathered during exposure to ultraviolet light.

### Statistical Analysis

Data were analyzed by mean ± SEM. Each experiment was duplicated more than three times. Two-tailed unpaired Student’s *t*-tests was applied to compare the data of two E groups. Statistically significant values: *P* < 0.05, *P* < 0.01, and *P* < 0.001 were, respectively, represented as ^∗^, ^∗∗^, ^∗∗∗^. “n.s.” indicates non-significant.

## Data Availability Statement

The original contributions presented in the study are included in the article/Supplementary Material, further inquiries can be directed to the corresponding author.

## Ethics Statement

The animal study was reviewed and approved by the Animal Research Committee of Zhejiang University.

## Author Contributions

H-YF designed and supervised the project. H-YF and L-RC wrote the manuscript. L-RC and J-CJ conducted the experiments. All authors contributed to the article and approved the submitted version.

## Conflict of Interest

The authors declare that the research was conducted in the absence of any commercial or financial relationships that could be construed as a potential conflict of interest.
