# Supplementary material for: Positive Feedback Stimulation of Ccnb1 and Mos mRNA Translation by MAPK Cascade During Mouse Oocyte Maturation
Source: Front Cell Dev Biol. 2020 Nov 13;8:609430. doi: 10.3389/fcell.2020.609430 (PMC7691486; doi:10.3389/fcell.2020.609430)
Supplement: Supplementary file 1 [file Table_1.DOCX]

**Supplementary Table 1. Antibody information**

| **Protein name** | **Manufacture**  **(catalogue number)** | **Applications**  **(working dilution)** |
| --- | --- | --- |
| **FITC-α-Tubulin** | Sigma (F2168) | WB (1:1000), IF (1:500) |
| **p-ERK1/2** | Cell Signaling (9101S) | WB (1:1000) |
| **FLAG** | Sigma (F3165) | WB (1:1000) |
| **CPEB1** | Proteintech (13274-1-AP) | WB (1:1000) |
| **cyclin B1** | Cell Signaling (4138) | WB (1:1000) |
| **MOS** | Abcam (171937) | WB (1:1000) |
| **DDB1** | Epitomics (3821-1) | WB (1:2000) |

**Supplementary Table 2. Primer sequences used in the poly(A) tail assay**

| **Gene name** | **Primer sequence** |
| --- | --- |
| ***Ccnb1_long 3_***_′_***_-UTR_-*FP** | 5′-GTGTGCTTTGAATTCTGACAGCCA-3′ |
| ***Ccnb1_long 3_***_′_***_-UTR_-*A0** | 5′-GCTTTCCACCAATAAATTTTATTC-3′ |
| ***Mos-*FP** | 5′- CATCGAGCCGATGTAGAGATAAGC-3′ |
